# Extensive Drug Resistance of Strong Biofilm-Producing *Acinetobacter baumannii* Strains Isolated from Infections and Colonization Hospitalized Patients in Southern Poland

**DOI:** 10.3390/pathogens12080975

**Published:** 2023-07-26

**Authors:** Tomasz Kasperski, Dorota Romaniszyn, Estera Jachowicz-Matczak, Monika Pomorska-Wesołowska, Jadwiga Wójkowska-Mach, Agnieszka Chmielarczyk

**Affiliations:** 1 Doctoral School of Medical and Health Sciences, Jagiellonian University Medical College, 31-008 Krakow, Poland; tomasz.kasperski@doctoral.uj.edu.pl; 2 Department of Microbiology, Faculty of Medicine, Jagiellonian University Medical College, Czysta 18 Street, 31-121 Cracow, Poland; d.romaniszyn@uj.edu.pl (D.R.); estera.jachowicz-matczak@uj.edu.pl (E.J.-M.); jadwiga.wojkowska-mach@uj.edu.pl (J.W.-M.); 3 Department of Microbiology, Analytical and Microbiological Laboratory of Ruda Slaska, KORLAB NZOZ, 41-703 Ruda Slaska, Poland; monikapw@op.pl

**Keywords:** *Acinetobacter baumannii*, biofilm, drug resistance

## Abstract

*Acinetobacter baumannii* (AB) is a bacterium that causes infections, particularly in immunocompromised patients. Treatment is challenging due to biofilm formation by AB strains, which hinders antibiotic effectiveness and promotes drug resistance. The aim of our study was to analyze the biofilm-producing capacity of AB isolates from various forms of infections in relation to biofilm-related genes and their drug resistance. We tested one hundred isolates for biofilm formation using the crystal violet microplate method. Drug resistance analyses were performed based on EUCAST and CLSI guidelines, and biofilm genes were detected using PCR. All tested strains were found to form biofilms, with 50% being ICU strains and 72% classified as strong biofilm producers. Among these, 87% were extensively drug-resistant (XDR) and 2% were extra-extensively drug-resistant (E-XDR). The most common gene set was *bap*, *bfm*S, *csu*E, and *omp*A, found in 57% of all isolates. Our research shows that, regardless of the form of infection, biofilm-forming strains can be expected among AB isolates. The emergence of E-XDR and XDR strains among non-ICU infections highlights the necessity for the rational use of antibiotics to stop or limit the further acquisition of drug resistance by *A. baumannii*.

## 1. Introduction

*Acinetobacter baumannii* (AB) is an opportunistic pathogen, dominant in Central and Southern Europe in healthcare-associated infections (HAIs). It is considered by the WHO as a critical-priority pathogen, for which there is an urgent need to search for new therapeutic solutions, primarily due to acquired resistance mechanisms [1,2]. It causes a variety of infections as pneumonia in ventilated patients, bacteremia, or urinary tract infections [3,4].

According to the European Antimicrobial Resistance Surveillance Network (EARS Net), the prevalence of Acinetobacter isolates resistant to at least one of the antimicrobial groups varies greatly according to country, ranging from below 1% to 98.6%. In 2021, 3 countries (Ireland, The Netherlands, and Norway) reported a prevalence of less than 1%, while in 25 European countries, it was above 50%—the highest level of resistant isolates is recorded in Eastern and Southern Europe, including Poland [5].

In Polish hospitals, especially in ICUs, the dominance of extensively drug-resistant (XDR) AB, defined as those strains that are susceptible to no more than two antimicrobial classes, has been noted for many years, and it is one of the major therapeutic problems associated with Gram-negative bacilli. The prevalence of AB isolates resistant to carbapenems in 2021 reached 82.7%, while 67% were resistant to carbapenems, fluoroquinolones, and aminoglycosides together [5,6,7,8].

Multidrug resistance in AB is associated with numerous mechanisms—enzymatic degradation, modification of antibiotics, reduction in membrane permeability, and increased efflux. Carbapenem resistance of AB is conferred by carbapenem-hydrolyzing class D oxacillinases (OXA): OXA-23-like, OXA-24/40-like, OXA-58-like, and intrinsic OXA-51-like. Permeability to beta-lactams, efflux pumps, and ISAba1 elements located upstream of the blaOXA-51-like gene also contributes to carbapenem resistance. Often, the production of carbapenemases coexists with overexpression of efflux pumps [9].

The connection between biofilms and antibiotic resistance is of a considerable interest to biomedical researchers. The ability to form a biofilm, which is possessed by a large percentage of AB strains (significantly higher than in the case of other Acinetobacter species [10]), is considered to be one of the main factors of virulence and also directly contributes to the antibiotic resistance of bacteria, increasing tolerance to drugs and acting as a barrier against the penetration of antimicrobial agents or altering their metabolism and action [11]. AB outside HAIs can also cause community-acquired infections, but, still, little is known about the main natural reservoirs of this pathogen [12]. Extra-hospital reservoirs of AB, such as natural habitat, animals, food, high-touch surfaces in cities, and the routes of transmission of this pathogen within the community and between the community and hospital environment, are being investigated. The presence of AB has been demonstrated both in the urban environment on frequently touched surfaces as well as soil, water, plants, or food of animal origin [13,14]. Among the strains isolated from food, biofilm-forming strains and multidrug-resistant strains were also found, which may be a potential reservoir of new genes of carbapenemases carried on plasmids [13,15,16].

In the hospital environment, biofilm formation promotes the long-term persistence of AB on abiotic surfaces [17]. Under unfavorable environmental conditions, AB cells in the biofilm can become dormant and metabolically inactive, allowing them to survive environmental stress [18]. The ability to form a biofilm is facilitating colonization of patients and, consequently, infection. Infections connected with biofilm-forming AB strains associated with medical devices, primarily in central venous catheter-related bloodstream infections (CVC-BSI), have been confirmed [19]. Very often, infections with biofilm-forming strains of AB are manifested by ventilator-associated pneumonia (VAP) [20]. The ability to increase environmental contamination, combined with the multidrug resistance of this microorganism, may lead not only to the development of infection and its severity but also to clonal spread, and this and result in outbreaks in hospital wards [21,22].

Biofilms, as organized multicellular communities of bacteria, are surrounded by self-produced exopolysaccharide matrices. The ability to form both biofilms and genes involved in this process has been studied extensively in recent years. The formation and development of the biofilm involve many virulence factors, such as the outer membrane protein A (OmpA), biofilm-associated protein (Bap), chaperon-usher pilus (Csu), extracellular exopolysaccharide (EPS), and two-component regulatory system (BfmS/BfmR) [10,17,23]. Csu pili are adhesive organelles and are required to induce in the initial adhering of a biofilm, promoting the maturation of the biofilm and maintaining the structure of the mature biofilm. The BfmR/BfmS system coordinates the gene expression from the Csu cluster [20]. The OmpA protein and the extracellular exopolysaccharide also act as adhesins. The Bap protein (biofilm-associated protein), in turn, is a key component of the mature biofilm and is involved in their various stages of formation.

The aim of our research was to investigate the biofilm-forming ability of AB strains recovered from bloodstream infections, pneumonia, skin and soft tissue infections, and colonizing patients in relation to the presence of biofilm-related genes and antimicrobial resistance.

## 2. Materials and Methods

### 2.1. Bacterial Isolates

The studied AB strains were isolated from clinical materials collected from patients hospitalized in 2019–2021 in 7 hospitals of the Silesian Province in Southern Poland (mainly Katowice, Sosnowiec, Knurów, Mysłowice) and 2 hospitals of the Opolskie Province (Opole, Głuchołazy) and from materials collected as part of screening tests at the University Hospital in Krakow. The collection was stored in the laboratory of the Department of Microbiology at −70 °C.

One hundred AB isolates were randomly selected applying the principle of one isolate from one patient, taking into account three clinical forms of infection, such as bloodstream infection (BSI), pneumonia (PNEU), skin and soft tissue infections (SSTIs), and colonization. Patients were hospitalized in ICU and non-ICU units (general surgery, orthopedics, neurology, internal medicine, palliative medicine). In this way, 25 isolates from BSI, 25 from PNEU, 25 from SSTIs, and 25 from patients without symptoms of infection (colonization) were collected.

The strains were collected in accordance with the consent of the bioethics committee of the Jagiellonian University KBET 1072.6120.274.2021 and KBET 1072.6120.2.2021.

Isolates were identified using automated systems (MALDI-TOFF identification; Maldi Biotyper, Bruker or MALDI TOF MS Vitek MS Home bioMérieux), depending on the hospital laboratory. In addition, the intensively producing biofilm reference strain, AB ATCC^®^ 19606™, was used in the study as a positive control in the experiment on the production of biofilm by bacterial isolates [24].

### 2.2. Antimicrobial Susceptibility Testing

Antibiotic susceptibility was determined based on the results of an automated system, MIDITECH Analyser v.12. The results were interpreted using the clinical breakpoints defined in the latest EUCAST guidelines—v. 13.0 [25] For cefoperazone/sulbactam, the interpretation was made based on the manufacturer’s instructions for the ATCC reference strain and ampicillin/sulbactam and piperacillin/tazobactam based on CLSI (Clinical and Laboratory Standards Institute) guidelines [26]. Antibiotic resistance to tigecycline was determined for all strains using the MIC Test Strip (TGC 0.016–256 mg/L; Liofilchem Diagnostic; Roseto degli Abruzzi, Italy). Results were interpreted in accordance with EUCAST recommendations using MIC for non-species-related breakpoints. The MIC for colistin was confirmed using the microdilution method (MIC STRIPPED PLATES COL; Diagnostics, Galanta, Slovakia), and the results were interpreted in line with the manufacturer’s instructions according to EUCAST v. 13.0. Based on the obtained results, the isolates were classified in terms of multidrug resistance as non-multidrug-resistant (nMDR), multidrug-resistant (MDR), extensively drug-resistant (XDR) [27], and E-XDR extra-extensively drug-resistant isolates. MDR strains were defined as those strains that were non-susceptible to one antimicrobial in at least three different antimicrobial classes. XDR strains were defined as those strains that were susceptible to no more than two antimicrobial classes [23]. E-XDR strains were defined as strains resistant to all antibiotics tested in this work. Strains showing intermediate susceptibility to any of the antibiotics were interpreted as non-susceptible and counted in the group of resistant strains.

The pattern of antimicrobial resistance was defined as the set of antibiotics to which at least two strains are resistant. For all strains, the multi-antibiotic-resistant (MAR) index was calculated. The MAR index is the number of antibiotics that an isolate is resistant to divided by the total number of antibiotics utilized in the study [28].

$$MAR\;Index=\frac{number\;of\;resistant\;antibiotics}{total\;number\;of\;antibiotics}$$

### 2.3. Detection of Carbapenemase Genes

The Genomic Mini AX Bacteria Kit (A&A Biotechnology, Gdansk, Poland) was used to extract genomic DNA from AB isolates following the manufacturer’s protocol. The concentration and purity of the isolated DNA was assessed using a Nano Drop Lite spectrophotometer (Thermo Fisher Scientific, Waltham, MA, USA). DNA extracted from pure cultures was stored at −20 °C for further study.

The most common carbapenemases genes in AB in Poland were detected: *bla*_OXA-23,_ *bla*_OXA-40_, *bla*_OXA-58_, and *bla*_NDM_ [7]. Detection of carbapenemasse genes was carried out according out following the protocol described by Cerezales et al. [29]. In the multiplex polymerase chain reaction (PCR), *bla*_OXA-23_ (718 bp), *bla*_OXA-40_ (413 bp), *bla*_NDM_ (517 bp), and *bla*_OXA-58_ (303 bp) genes were identified (Table 1). PCR amplification was performed using the Color OptiTaq PCR Master Mix (EURx Ltd., Gdańsk, Poland) in a final volume of 25 μL, with a final primer concentration of 0.1 μM for each primer. Bacterial DNA functioned as the template. PCR was conducted with an initial denaturation step of 3 min at 94 °C, followed by 30 cycles of 30 s at 94 °C, 15 s at 58 °C, and 1 min at 72 °C for amplification and a final extension step of 5 min at 72 °C. PCR products were analyzed via gel electrophoresis.

### 2.4. Assessment of Biofilm Formation

#### 2.4.1. Quantification of Biofilm Formation Assessment

Quantification of biofilm formation was performed as previously described by Stepanovic [30] with some modifications. Contrary to the original work, plates with a lower number of wells (24 wells) were used to increase the area of biofilm formation, and absorbance was measured by dry staining and fixating of the biofilm formed and measuring its thickness at 225 points of each well using a TECAN Infinite^®^ 200 plate reader PRO (Tecan Trading AG, Männedorf, Switzerland).

Briefly, a bacterial suspension of approx. 0.5 MacFarland (1.5 × 10^8^ CFU/mL) in saline was prepared from colonies on Tryptic Soy Agar (TSA) plates (TSA, Becton Dickinson, Franklin Lakes, NJ, USA). Twenty microliters of the prepared bacterial suspension was mixed with 1980 μL of Tryptic Soy Broth (TSB) (TSB, Becton Dickinson, Franklin Lakes, NJ, USA) and applied to 24-well flat-bottomed plates (Costar^®^ Corning HTL SA, Warsaw, Poland) yielding a titer of approximately 1.5 × 10^6^ CFU/mL.

The plate was incubated at 37 °C without shaking for 20 h. Liquid medium alone (TSB) was used as a negative control. ATCC 19606 strain was used as a positive control. After incubation, the medium with unbound cells was gently removed, then carefully rinsed three times with PBS and fixed with methanol for 30 s. The plate was dried overnight at 37 °C upside down, and then the biofilm was stained with crystal violet (1000 µL) for ~15 min at room temperature. After this time, the dye was poured off and rinsed with distilled water until the water in the wells was colorless. Again, it was dried overnight at 37 °C upside down. After the plate was completely dried, the optical density absorbance was measured at 570 nm on the surface of the biofilm formed at 225 points of each well. For each strain, two replicates were analyzed to detect the biofilm formation ability.

Using the i-control software (ver. 2.0.10.0), the mean optical density (OD) and standard deviation (SD) values were calculated for each test isolate and all replicates. The cut-off point (ODc) was calculated using the following formula: ODc = mean OD of the negative control + (3 × standard deviation (SD) of the negative control). The averaged OD value of the tested isolates was reduced by the ODc value. The ODc value was calculated for each 12-well plate separately.

The strains were categorized according to Stepanović [30] into four categories: 0: non-biofilm producers (OD variable below cut-off), 1: poor biofilm producers (OD variable ≤ 2 × cut-off), 2: moderate biofilm producers (OD variable) from 2 × to 4 × cut-off), 3: strong biofilm producers (>4 × cut-off).

#### 2.4.2. Detection of Biofilm-Associated Genes

Genomic DNA was extracted as described above.

Detection of biofilm-associated genes was carried out in PCR. The genes *bap*, *csu*E, *omp*A, *bfm*S, and *esp*A were identified (Table 2). PCR amplification was performed using Color OptiTaq PCR Master Mix (EURx Ltd., Gdańsk, Poland) in a final volume of 25 μL, with a final primer concentration of 0.1 μM for each primer. Bacterial DNA functioned as a template. For genes *omp*A, *bfm*S, and *esp*A, PCR was performed with an initial denaturation step of 5 min at 94 °C, followed by 35 cycles of 1 min at 94 °C, 1 min at 55 °C, and 45 s at 72 °C for amplification and a final extension step of 5 min at 72 °C. PCR products were analyzed using gel electrophoresis. For *bap* and *csu*E, PCR was performed with an initial denaturation step of 5 min at 96 °C, followed by 35 cycles of 1 min at 96 °C, 1 min at 56.5 °C for *bap*, and 57 °C for *csu*E and 1 min at 72 °C for amplification and a final extension step of 10 min at 72 °C. PCR products were analyzed using gel electrophoresis.

### 2.5. Statistical Analysis

In statistical analyses, the determination of significant differences between the groups of isolates with low and moderate biofilm production and the group with intensive biofilm production and the clinical types of infection or colonization (BSI, PNEU, SSTI, colonization) was demonstrated in the cross-analysis in the Fisher–Freeman–Halton exact test with two-sided exact significance *p* ≤ 0.05. The differences between the prevalence of resistant isolates and the ability to form a biofilm were analyzed based on the Pearson Chi-square test. The Fisher–Freeman–Halton test was used to show the variability between resistant strains isolated from different clinical forms of infection. The univariate analysis of variance (ANOVA) was used to find significance between associations for the different combination of the biofilm genes and the ODs and significance between the MDR or XDR and the type of the produced biofilm. The results were considered significant at *p* ≤ 0.05.

## 3. Results

### 3.1. Bacterial Isolates

A total of 100 AB isolates were tested following the principle of 1 strain per patient, including 25 from PNEU, 25 from SSTI, 25 from BSI, and 25 from colonization. Fifty-three percent of the strains came from ICU patients, including all colonization strains from ICU, 56% from PNEU infections, and 44% from BSI infections. In contrast, only 12% of SSTIs were from ICU. The morbidity and distribution of AB in ICU and non-ICU isolates are shown in Table 3.

### 3.2. Antimicrobial Susceptibility Testing

The highest rate of resistance was for cefoperazone/sulbactam (95%) and the lowest was for colistin (8%). Strains resistant to both carbapenems (imipenem and meropenem) were 69%, and 45% were resistant to the carbapenems, fluoroquinolones, and aminoglycosides tested. A significant difference was found for resistance to imipenem (*p* = 0.023) and meropenem (*p* = 0.023) between strains from the colonization and SSTI groups and for gentamicin between the colonization and SSTI; In the BSI and PNEU groups (*p* = 0.024, *p* = 0.001, *p* = 0.048 respectively), no significant differences were observed in the case of other antimicrobials and the other groups of strains (Table 4).

The most common pattern of resistance among all AB strains was resistance to all tested antibiotics except colistin (37%; n = 37) (Table 5). Among the tested isolates, 75% were classified as XDR and 2% as E-XDR. While 9.4% of the ICU strains were not multidrug resistant, nearly 87% of the strains were classified as XDR and E-XDR (Table 6).

### 3.3. Selected Carbapenemases Genes

The most frequently detected gene among all AB strains was *bla*_OXA-40_ (42%); considering the clinical form of infection, it was detected in 72% of strains from SSTI and 56% of strains from colonization. In turn, in BSI strains, the *bla*_OXA-23_ gene was found more often (44%). Three strains isolated from patient colonization had the *bla*_NDM_ gene (Table 7). The *Bla*_NDM_ gene was carried together with the *bla*_OXA-23_ gene. Two strains (from PNEU and colonization) had a combination of OXA-51/ISAba-I upstream. None of the isolates carried the *bla*_OXA-58_ gene. Further, 17 of the 31 strains that were carbapenem-sensitive in the phenotypic study, nevertheless, had one of the oxacillinase genes.

### 3.4. Quantitative Biofilm Formation Assessment

**Figure 1:**
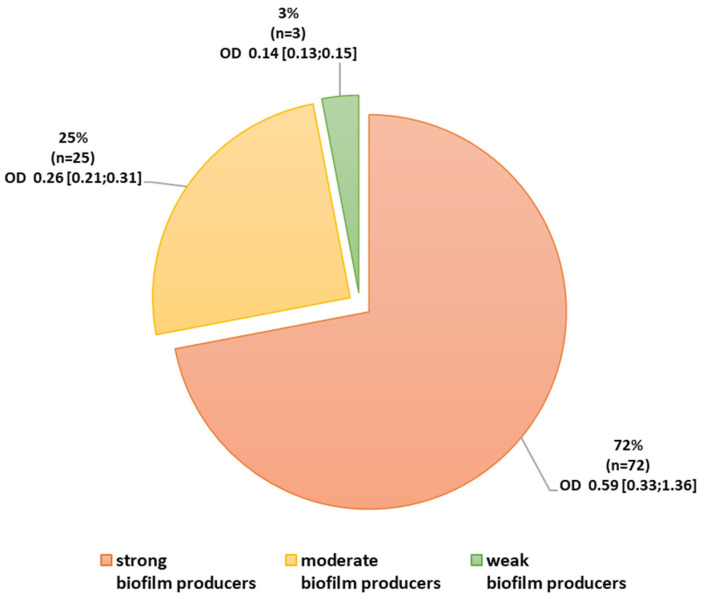
Ability to form a biofilm among *Acinetobacter baumannii* isolates. Legend: OD absorbance value: mean (minimum; maximum).

Quantification of biofilm production showed that all tested isolates (n = 100) produce a biofilm: 3% were classified as weak producers of biofilm, 25% as moderate, and 72% as strong (Figure 1). Among the ICU strains, 77% (n = 41) were classified as strong biofilm producers and 66% (n = 31) from non-ICU (Table 8).

### 3.5. Correlation between the Ability to Biofilm Formation and Resistance among Strains with Different Clinical Forms of Infection or Colonization

A group of strains characterized by weak and moderate biofilm production was combined for statistical analyses, due to the small number of strains in the group of weak biofilm producers (n = 3 and n = 25).

**Figure 2:**
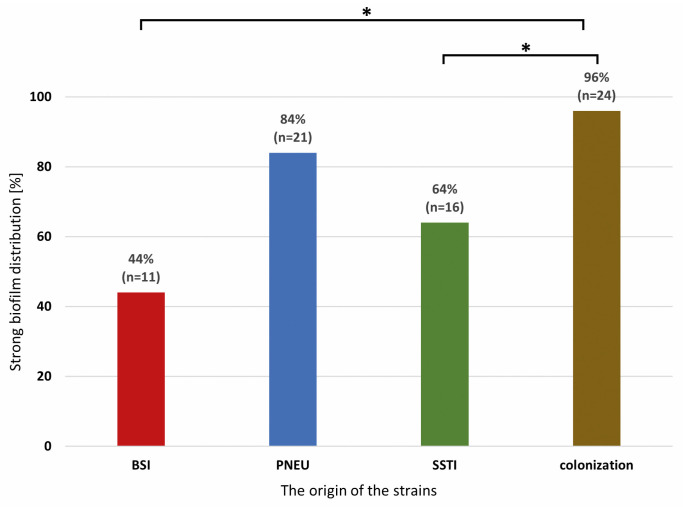
Distribution of strong biofilm-producing *Acinetobacter baumannii* isolates from various clinical forms of infection and colonization. Legend: BSI—bloodstream infection, PNEU—pneumonia, SSTI—skin and soft tissue infection, colonization; significance is marked with an asterisk * *p* < 0.001.

Most of the strains strongly producing a biofilm were in the group of colonization and PNEU. Significant differences (two-sided exact significance *p* < 0.001) were found between the type of infection and the ability to form a biofilm among the isolates from the colonization group in relation to BSI and SSTI. No differences were found between isolates from different clinical forms of infection (Figure 2).

The correlation between biofilm formation and resistance to specific antibiotics was also analyzed. All colistin-resistant strains (n = 8) were strong biofilm producers. A statistically significant difference (*p* = 0.012) was found between the number of isolates resistant to gentamicin in the group of strong biofilm producers (47.2%; n = 34) and in the group of weak and moderate biofilm producers (75%; n = 21).

**Figure 3:**
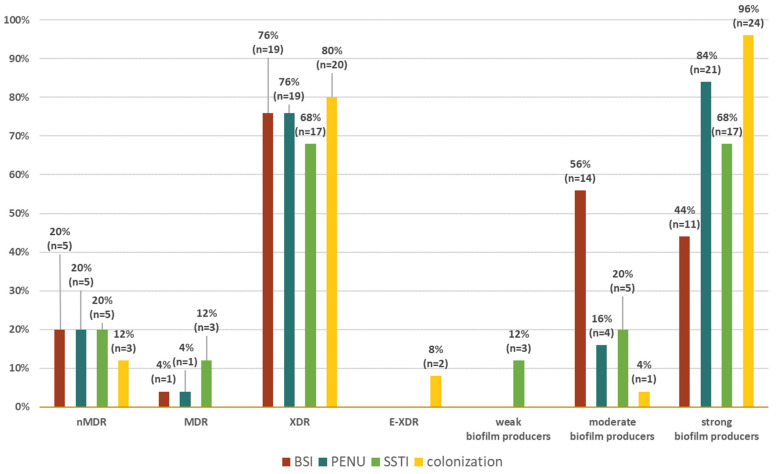
Distribution of clinical forms of infection among nMDR, MDR, XDR, and E-XDR strains and weak, moderate, and strong biofilm-producing *Acinetobacter baumannii* strains. Legend: BSI—bloodstream infection, PNEU—pneumonia, SSTI—skin and soft tissue infection, nMDR—no multidrug resistant, MDR—multidrug resistant, XDR—extensively drug resistant, E-XDR—extra-extensively drug resistant.

The distribution of resistance in relation to resistance groups (nMDR, MDR, XDR, and E-XDR) and groups of different biofilm producers (strong, moderate, weak) in different groups of origin of the strains (BSI, PNEU, SSTI, colonization) is shown in a radar chart (Figure 3). There was no significant correlation between the resistance group and the type of biofilm producer.

AB strains, regardless of the origin of the strains, were characterized by a similar distribution of XDR resistance. Among SSTI isolates, it was 68% (n = 17), BSI 76% (n = 19) and PNEU 76% (n = 19), and colonization was 80% (n = 20).

All weak biofilm-producing strains (n = 3) were isolated from SSTI, while moderate biofilm-producing strains were present in all other groups, and most of them (56%; n = 14) were isolated from BSI.

### 3.6. Selected Biofilm-Associated Genes

Among the tested isolates, the most frequently detected gene was *omp*A 99%, while the least frequently detected was the *eps*A gene 26%.

The most common characteristic genotype was *bap*/*bfm*S/*csu*E/*omp*A, observed in 57% of isolates. Among them, 66.67% (n = 38) belonged to the group of strong biofilm producers.

The set of all five tested genes, *bap*/*bfm*S/*csu*E/*omp*A/*eps*A, was less frequent (19%), but 78.9% (n = 15) of isolates with this set were strong producers of a biofilm. The third most common set of genes, *bfm*S/*csu*E/*omp*A, present in 12% of isolates, concerned 74% (n = 9) of strong biofilm producers.

**Figure 4:**
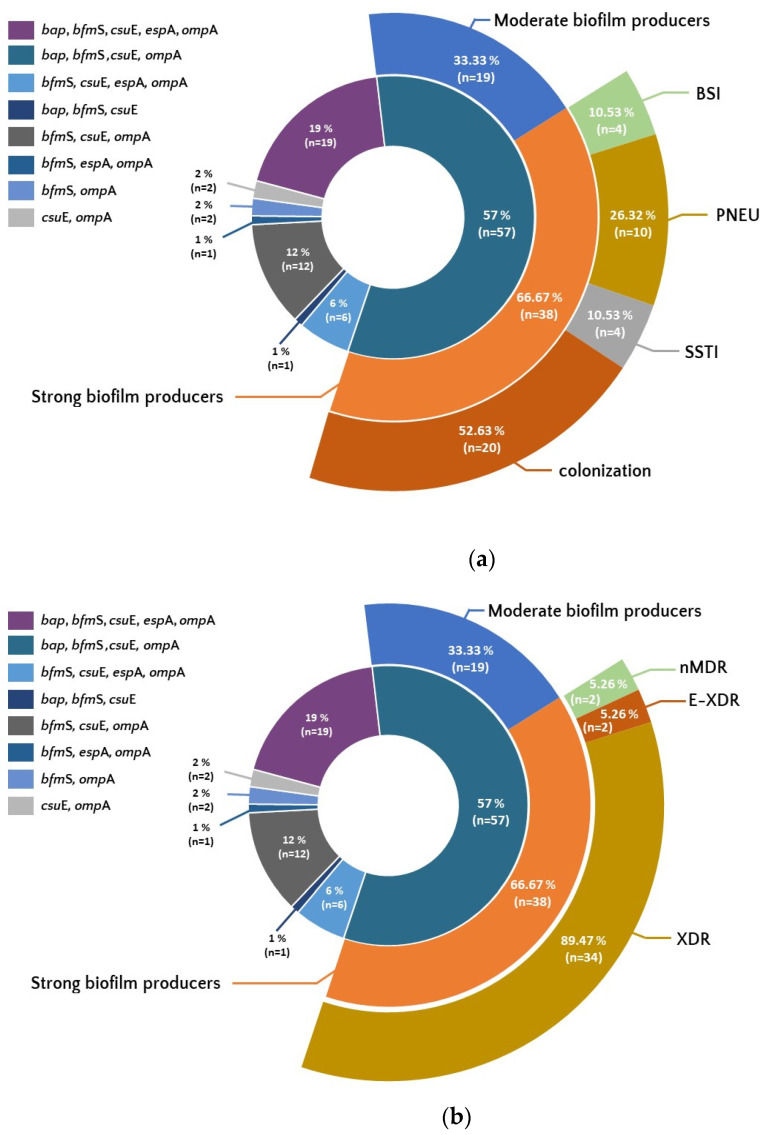
Occurrence of set of genes associated with biofilm formation among isolates showing differences in biofilm production, resistance (**b**), and from different forms of clinical infection and colonization (**a**). Legend: BSI—bloodstream infection, PNEU—pneumonia, SSTI—skin and soft tissue infection, nMDR—no multidrug resistant, MDR—multidrug resistant, XDR—extensively drug resistant, E-XDR—extra-extensively drug resistant.

In the group of the most characteristic genotype *bap*/*bfm*S/*csu*E/*omp*A, more than half of the isolates were isolates from the colonization group (52.63%; n = 20). The same genotype was the least frequent in the group of isolates from BSI (10.53%; n = 4). Moreover, the same set of genes was found among all E-XDR strains (5.26%; n = 2) and in 89.47% (n = 34) of XDR strains (Figure 4a,b).

No significant association was found between different combinations of biofilm genes and OD biofilms in the biofilm assay for strong biofilm formers.

## 4. Discussion

In our study, the vast majority of AB strains (72%) strongly produced a biofilm and were characterized by the presence of four genes associated with biofilm formation (*bap*, *bfm*S, *csu*E, *omp*A). Also, most belonged to the XDR group (75%) and were resistant to imipenem and meropenem (69%), noting that only half of the isolates were from ICU. It is alarming to note eight colistin-resistant strains that were isolated during screening tests from asymptomatic patients.

The WHO has listed carbapenem-resistant AB as a critical-priority pathogen among those bacteria that require the research and development of new drugs [1]. The situation in Europe for resistant ABs is not uniform as it is the pathogen with the greatest cross-country distribution. By far, the highest prevalence of strains resistant to carbapenems, as well as to three groups of drugs combined, is found in Southern and Eastern Europe, and it reaches over 90% in Greece, Romania, and the Balkan countries (the current European average is 39.9%, and it has increased by 6.8% compared to the average from 2017).

ECDC and WHO data for Poland indicate that the number of ABs resistant to fluoroquinolones, aminoglycosides, and carbapenems together increased from 59.5% in 2017 to 67.0% (2021) [5]. AB also accounted for 55% of all bacterial isolates from the ICU [5]. Our current study indicates that the situation seems to be worse than in our previous studies, where we recorded 80 and 86% of XDR, but nearly 80% of isolates came from the ICU (we tested only 53% ICU isolates) [7,35]. Other authors from Poland reported 76.5% XDR among ICU isolates [36]; our current study shows 86.8% XDR (including E-XDR) considering only ICU strains. Additionally, we found two strains resistant to all antibiotics tested in this work (E-XDR). So far, no data have been published in Poland indicating the presence of AB isolates in PDR or E-XDR. These strains originated from the colonization of a patient but, unfortunately, we do not have data on whether they later contributed to infection in these patients. Our strains were overwhelmingly sensitive to colistin (92%) but also to ampicillin-sulbactam (69%). We did not test new drugs, such as cefiderocol and ervacycline, and we reported only 19% susceptibility to tigecycline. In recent years, isolates resistant to all drugs (PDR) or to the vast majority of subjects, including colistin (E-XDR), have appeared mainly in Asian countries [37,38].

The predominant carbapenemases genes in the studied AB strains were *bla*_OXA-40_ (42%) and *bla*_OXA-23_ (26%), which confirms our previous studies as well as other reports from Poland [7,39,40]. The *Bla*_NDM_ gene was also detected in three strains from colonized patients. Slightly over 26% of carbamenem-resistant strains did not carry any of the oxacillinase genes tested; however, carbapenem-hydrolyzing class D β-lactamases (CHLD) have so many types of oxacillinase that it is possible that they have other genes not detected in our study. Resistance in AB also results from non-enzymatic mechanisms of resistance, e.g., activity of efflux pumps. One family of efflux pumps is the RND family. This efflux pump is also involved in biofilm formation and maturation. Yoon et al. [41] showed that in mutants in RND pump genes, biofilm formation is significantly reduced compared to wild-type strains. This could explain the association between multidrug resistance and strong biofilm production. The antibiotic resistance of bacteria growing in the biofilm will be higher, even when the strains growing in the form of planktonic cells do not have the acquired resistance mechanisms [23,42]. Kim et al. [43] indicate, however, that correlations between efflux pump genes and biofilm formation and resistance are not always clear-cut, as, in their studies, increased efflux activity occurred among poor biofilm producers, although it also correlated with resistance to tigecycline and cefotaxim.

The vast majority of studied strains tested in this publication were biofilm-producing strains, including strong producers, which accounted for 72%. In studies, where, like in ours, the association between biofilm formation and antimicrobial resistance was checked, regardless of the mechanism, a positive correlation was found much more often; strains strongly producing biofilms were characterized by higher resistance, primarily to antibiotics from the group of B-lactams and aminoglycosides, or the XDR phenotype in general [10,44,45,46,47].

In our earlier research [48], the vast majority of strains produced biofilms of nearly 82%, but most were included in the moderate biofilm producers group. In this study, we observed that a large number of biofilm-producing strains were susceptible to amikacin or tobramycin, and these were strains isolated from ICU patients [48]. These findings showed how important it is to take into account other factors, such as the types of hospital units, when describing the relationship between biofilms and resistance.

In our current study, most of the strains were classified as strong biofilm producers. We introduced—in relation to previous experiments—a modification of the study and assessment of biofilm intensity; namely, we read the absorbance of crystal violet on the surface of the created biofilm instead of in the solution, and, additionally, the measurement was made at 225 points. This approach allows for the classification of strains into a given group of biofilm producers with high accuracy. We showed a significant relationship in strains isolated from patients without clinical symptoms of infection and strains isolated from patients with pneumonia and between AB strong biofilm producers. Most of these patients were hospitalized in ICUs. The vast majority of strains strongly producing biofilms were XDR type, including all of them being resistant to colistin. The only antibiotic for which those isolates were more likely to produce less biofilm was gentamicin.

AB survival on often-touched surfaces may have an impact on the spread of AB strains in hospital environments. It is extremely important to understand the impact of biofilm formation and antibiotic resistance on AB survival in the hospital environment. Greene et al. [18] indicated interesting differences between clinical and environmental strains; in the case of the latter, the ability to form a biofilm is critical for the survival of the strains, while in the case of clinical strains, the MDR phenotype is more important. This study demonstrates a trade-off between antibiotic resistance and desiccation tolerance in hospital strains [18]. In his research, Qi et al. showed that a strong ability to form a biofilm can be a mechanism that allows bacteria to survive better, especially in the case of isolates with a sufficiently high level of resistance [49]. Our research, in turn, seems to confirm the thesis that AB-HAIs in Polish ICUs are dominated by strains that are characterized by both high enzymatic drug resistance and high virulence (including biofilm formation). Ababneh et al. showed that in hospital environments, frequently touched surfaces, especially in ICUs for adults and children, are contaminated with AB strains with the XDR phenotype. The source of these strains may be patients even with a distant history of infections with multidrug-resistant AB strains [50].

The presence of genes that are mainly associated with biofilm production was confirmed in both strong and moderate biofilm producers. The strong biofilm-producing AB represents 70% of the most common set of genes (*bap*, *bfm*S, *csu*E and *omp*A), from which 87.2% are XDR. Other studies report a high frequency of *csu*E, *bap,* and *omp*A genes [10,17,51]. The Csu and Bap systems significantly increase adherence to the cell line, and Bap is also involved in the formation and maintenance of mature biofilms. The least common gene in our research was *eps*A, which codes for extracellular exopolysaccharide, which is consistent with the reports of Thummeepak et al. and inconsistent with Zeighami et al. [10,17]. It is also believed that *omp*A and *bap* gene products may contribute to the drug-resistant AB phenotype, especially OmpA, an outer membrane porin.

The resistance of AB to most antibiotics and the fact that it persists in the hospital environment for a long time cause a high risk of transmission of resistant (E-XDR and XDR) and highly biofilm-forming strains. These strains pose a serious threat to patients and a challenge for physicians in treatment [52,53]. In the used combination therapy, some combinations of drugs also showed significant inhibition of Acinetobacter biofilm, which may be an advantage of using the combination therapy. Such activities are demonstrated by, among others, imipenem-rifampicin, colistin-rifampicin, meropenem-sulbactam, and tigecycline-sulbactam [54,55]. Biofilm inhibitors in combination with antibiotics, such as zinc lactate or furanone with carbapenems, tigecycline, or polymyxin B, are also being tested. Such combinations work synergistically in in vitro studies [56].

New strategies are also being sought to combat biofilm-forming and multidrug-resistant strains, such as new antibiotics, e.g., synthetic lipopeptides [57], natural products, e.g., myrtenol, which also suppresses biofilm-forming genes [58], therapy with bacteriophages alone or in combination with an antibiotic [59,60]. High hopes are associated with cefiderocol, a new synthetic, siderophore cephalosporin. Bassetti et al. [61] systematically reviewed papers and concluded that cefiderocol is a promising and safe antibiotic option for the treatment of patients with carbapenem-resistant AB infections. Cefiderocol was approved for the treatment of infections caused by aerobic Gram-negative bacteria in adults with limited treatment options by the European Medicines Agency (EMA) in November 2019 and in Poland in March 2021 [62,63].

Regardless of the search for new solutions in the fight against multidrug-resistant AB, infection prevention and control are important in reducing AB infections. These include hand hygiene, environmental cleaning, provision and appropriate use of personal protective equipment, appropriate training of healthcare staff, and promotion of antimicrobial stewardship programs [5,6,64]. In the case of carbapenem-resistant AB strains, it is difficult to introduce surveillance of HAIs similar to the surveillance of carbapenem-resistant Enterobacterales (CRE) because the screening tests for Enterobacterales-specific carbapenemases are based on rapid cassette tests detecting KPC, NDM, VIM, IMP, and OXA-48 carbapenemases. The variety of types of carbapenemases in AB means that there are no similar tests for AB.

Current research on biofilm-forming AB, including ours, focuses mainly on the assessment of biofilm formation and its impact on bacterial resistance and survival in the environment; however, research on agents that destroy biofilms and interact with antibiotic therapy is also needed. Therefore, in our future studies, we plan to research, among others, the influence of bacteriophages on biofilms.

### Limitations

In this study, we did not detect the presence of efflux pumps from the RND family in the tested strains. Also, the effectiveness of cefiderocol on the tested strains was not tested. Strain genotyping was not performed. We have no information about the type of resistance to colistin (high-level resistance, heteroresistance, stable, or reversible) and about the association of BSIs when using catheters.

## Figures and Tables

**Table 1:** Primers used in detection of the carbapenemases genes in *Acinetobacter baumannii*.

Detected Genes	Primer Sequences (5′-3′) ^1^	Product Size (bp)	Annealing Temperature	Reference
*bla*_OXA-23_	F: TCTGGTTGTACGGTTCAGCA	718	58 °C	[29]
	R: GCATTTCTGACCGCATTTCC			
*bla*_OXA-40_	F: GCATTGTCAGCAGTTCCAGT	402	58 °C	[29]
	R: AGAACCAGACATTCCTTCTTTCA			
*bla*_NDM_	F: GTTTGATCGTCAGGGATGGC	517	58 °C	[29]
	R: CTCATCACGATCATGCTGGC			
*bla*_OXA-58_	F: ATCAAGAATTGGCACGTCGT	303	58 °C	[29]
	R: CCACATACCAACCCACTTGC			

^1^ F—forward; R—reverse.

**Table 2:** Primers used in detection of genes associated with the biofilm formation in *Acinetobacter baumannii*.

Detected Genes	Primer Sequences (5′-3′) ^1^	Product Size (bp)	Annealing Temperature	Reference
*bap*	F: TACTTCCAATCCAATGCTAGGGAGGGTACCAATGCAG	1225	56.5 °C	[31]
	R: TTATCCACTTCCAATGATCAGCAACCAAACCGCTAC			
*csu*E	F: ATGCATGTTCTCTGGACTGATGTTGAC	976	57 °C	[32]
	R: CGACTTGTACCGTGACCGTATCTTGATAAG			
*omp*A	F: CGCTTCTGCTGGTGCTGAAT	531	55 °C	[33]
	R: CGTGCAGTAGCGTTAGGGTA			
*bfm*S	F: TTGCTCGAACTTCCAATTTATTATAC	1368	55 °C	[34]
	R: TTATGCAGGTGCTTTTTTATTGGTC			
*esp*A	F: AGCAAGTGGTTATCCAATCG	451	55 °C	[33]
	R: ACCAGACTCACCCATTACAT			

^1^ F—forward; R—reverse.

**Table 3:** Morbidity and distribution of *Acinetobacter baumannii* in ICU and non-ICU isolates.

The Origin of the Strains	Morbidity			Distribution of Isolates		
	ICU (n = 53) No. (%)	Non-ICU (n = 47) No. (%)	Total No. (%)	ICU (n = 53) No. (%)	Non-ICU (n = 47) No. (%)	Total No. (%)
BSI	11 (20.7)	14 (29.8)	25 (25)	11 (44)	14 (56)	25 (100)
PNEU	14 (26.4)	11 (23.4)	25 (25)	14 (56)	11 (44)	25 (100)
SSTI	3 (5.7)	22 (46.8)	25 (25)	3 (12)	22 (88)	25 (100)
colonization	25 (47.2)	0 (0)	25 (25)	25 (100)	0 (0)	25 (100)
Total	53 (100)	47 (100)	100 (100)	53 (53)	47 (47)	100 (100)

Legend: ICU—intensive care unit, BSI—bloodstream infection, PNEU—pneumonia, SSTI—skin and soft tissue infection.

**Table 4:** Range of AMR of *Acinetobacter baumannii* isolates according to clinical form of infections and colonization.

Antibiotic Classes	Antimicrobial	Resistant Isolate; Number (%)				
		Total	The Origin of the Strains			
			BSI	PNEU	SSTI	Colonization
Penicillin	ampicillin/sulbactam piperacillin/tazobactam	65 (65%) 86 (86%)	16 (64%) 21 (84%)	17 (68%) 21 (84%)	12 (48%) 22 (88%)	20 (80%) 22 (88%)
Cephalosporins	cefoperazone/sulbactam	95 (95%)	23 (92%)	24 (96%)	23 (92%)	25 (100%)
Carbapenems	Imipenem * meropenem *	69 (69%) 69 (69%)	18 (72%) 18 (72%)	17 (68%) 17 (68%)	12 (48%) 12 (48%)	22 (88%) 22 (88%)
Fluoroquinolones	ciprofloxacin levofloxacin	87 (87%) 80 (80%)	23 (92%) 19 (76%)	23 (92%) 21 (82%)	18 (68%) 18 (72%)	22 (88%) 22 (88%)
Aminoglycosides	amikacin gentamycin ** tobramycin	69 (69%) 55 (55%) 70 (70%)	19 (76%) 19 (76%) 17 (68%)	18 (72%) 14 (56%) 19 (76%)	14 (56%) 15 (60%) 16 (64%)	18 (72%) 7 (28%) 18 (72%)
Tetracyclines	tigecycline	71 (71%)	18 (72%)	19 (76%)	15 (60%)	19 (76%)
Miscellaneous agents	colistin trimethoprim/sulfamethoxazole	8 (8%) 77 (77%)	0 (0%) 20 (80%)	0 (0%) 19 (76%)	0 (0%) 16 (64%)	8 (32%) 22 (88%)

Legend: BSI—bloodstream infection, PNEU—pneumonia, SSTI—skin and soft tissue infection. * A significant difference (*p* = 0.023) was found for resistance to imipenem and meropenem between strains from the colonization and SSTI (*p* = 0.023) groups. ** A significant difference was found for resistance to gentamicin (*p* = 0.007) between the colonization and SSTI (*p* = 0.024), colonization and BSI (*p* = 0.001) and colonization and PNEU (*p* = 0.048) groups.

**Table 5:** Antimicrobial resistance patterns of *Acinetobacter baumannii* isolates found in two or more strains.

Antibiotic Patterns *	No. of Isolates	MAR Index
SAM, TZP, SCF, IMP, MEM, CIP, LEV, AMI, GEN, TN, TIG, SXT	37	0.92
SAM, TZP, SCF, IMP, MEM, CIP, LEV, AMI, TN, TIG, SXT	10	0.85
TZP, SCF, IMP, MEM, CIP, LEV, AMI, GEN, TN, TIG, SXT	5	0.85
SCF	4	0.08
SCF, CIP	4	0.15
SAM, TZP, SCF, IMP, MEM, CIP, LEV, AMI, TN, TIG, SXT, CL	3	0.92
CIP	2	0.08
SAM, TZP, SCF, CIP, LEV, AMI, TN, TIG, SXT	2	0.69
SAM, TZP, SCF, CIP, LEV, GEN, TIG, SXT	2	0.62
SAM, TZP, SCF, IMP, MEM, CIP, LEV, AMI, GEN, TN, TIG, SXT, CL	2	1.00
SAM, TZP, SCF, IMP, MEM, CIP, LEV, AMI, TN, SXT	2	0.77
SAM, TZP, SCF, IMP, MEM, CIP, LEV, TIG, SXT, CL	2	0.77
TZP	2	0.08
TZP, SCF	2	0.15

Legend: SAM—ampicillin/sulbactam; TZP—piperacillin/tazobactam; SCF—cefoperazone/sulbactam; IMP—imipenem; MEM—meropenem; CIP—ciprofloxacin; LEV—levofloxacin; AMI—amikacin; GEN—gentamicin; TN—tobramycin; TIG—tigecycline; SXT—trimethoprim/sulfamethoxazole; CL—colistin; * the pattern of antimicrobial resistance was defined as the set of antibiotics to which strains are resistant

**Table 6:** Antimicrobial resistance groups of *Acinetobacter baumannii* in ICU and non-ICU isolates.

Group of Resistance	Morbidity			Distribution of Isolates		
	ICU (n = 53) No. (%)	Non-ICU (n = 47) No. (%)	Total No. (%)	ICU (n = 53) No. (%)	Non-ICU (n = 47) No. (%)	Total No. (%)
nMDR	5 (9.4)	13 (27.6)	18 (18)	5 (27.8)	13 (72.2)	18 (100)
MDR	2 (3.8)	3 (6.4)	5 (5)	2 (40)	3 (60)	5 (100)
XDR	44 (83)	31 (65.9)	75 (75)	44 (58.7)	31 (41.3)	75 (100)
E-XDR	2 (3.8)	0 (0)	2 (2)	2 (100)	0 (0)	2 (100)
Total	53 (100)	47 (100)	100 (100)	53 (53)	47 (47)	100 (100)

**Table 7:** Presence of selected carbapenemase genes among the *Acinetobacter baumannii* isolates according to type of unit, clinical form of infection, and group of resistance.

	*bla*_OXA-23_ N (%)	*bla*_OXA-40_ N (%)	*bla*_NDM_ N (%)	None of the Tested Genes N (%)
Type of unit (No.)				
Non-ICU (47).	13 (27.6)	23 (48.9)	0 (0)	13 (27.6)
ICU (53)	13 (24.5)	19 (35.8)	3 (5.7)	19 (35.8)
The origin of the strains (No.)				
BSI (25)	11 (44)	5 (20)	0 (0)	9 (36)
PNEU (25)	7 (28)	5 (20)	0 (0)	13 (52)
SSTI (25)	3 (12)	18 (72)	0 (0)	4 (16)
Colonization (25)	5 (20)	14 (56)	3 (12)	6 (24)
Group of resistance (No.)				
nMDR (18)	2 (11.1)	7 (38.9)	0 (0)	9 (50)
MDR (5)	0 (0)	2 (40)	0 (0)	3 (60)
XDR (75)	24 (32)	31 (41.3)	3 (4)	20 (26.7)
E-XDR (2)	0 (0)	2 (100)	0 (0)	0 (0)
Carbanenems resistant strains (69)	21 (30.4)	31 (44.9)	3 (4.3)	18 (26.1)
Total (100)	26 (26)	42 (42)	3 (3)	32 (32)

Legend: ICU—intensive care unit, BSI—bloodstream infection, PNEU—pneumonia, SSTI—skin and soft tissue infection, nMDR—no multidrug resistant, MDR—multidrug resistant, XDR—extensively drug resistant, E-XDR—extra extensively drug resistant.

**Table 8:** Biofilm production *Acinetobacter baumannii* in ICU and non-ICU isolates.

Group of Biofilm Producers	Morbidity			Distribution		
	ICU (n = 53) No. (%)	Non-ICU (n = 47) No. (%)	Total No. (%)	ICU (n = 53) No. (%)	Non-ICU (n = 47) No. (%)	Total No. (%)
Weak + moderate biofilm producers	12 (22.6)	16 (34)	28 (28)	12 (42.8)	16 (57.2)	28 (100)
Strong biofilm producers	41 (77)	31 (66)	72 (72)	41 (56.9)	31 (43)	72 (100)
Total	53 (100)	47 (100)	100 (100)	53 (53)	47 (47)	100 (100)

Legend: ICU—intensive care unit, weak biofilm producers (n = 3) and moderate biofilm producer (n = 25).

## Data Availability

Not applicable.
